# The recurrent pathogenic Pro890Leu substitution in CLTC causes a generalized defect in synaptic transmission in *Caenorhabditis elegans*

**DOI:** 10.3389/fnmol.2023.1170061

**Published:** 2023-05-31

**Authors:** Luca Pannone, Valentina Muto, Francesca Nardecchia, Martina Di Rocco, Emilia Marchei, Federica Tosato, Stefania Petrini, Giada Onorato, Enrico Lanza, Lucia Bertuccini, Filippo Manti, Viola Folli, Serena Galosi, Elia Di Schiavi, Vincenzo Leuzzi, Marco Tartaglia, Simone Martinelli

**Affiliations:** ^1^Molecular Genetics and Functional Genomics, Ospedale Pediatrico Bambino Gesù, IRCCS, Rome, Italy; ^2^Department of Human Neuroscience, “Sapienza” University of Rome, Rome, Italy; ^3^Department of Oncology and Molecular Medicine, Istituto Superiore di Sanità, Rome, Italy; ^4^National Centre on Addiction and Doping, Istituto Superiore di Sanità, Rome, Italy; ^5^Confocal Microscopy Core Facility, Ospedale Pediatrico Bambino Gesù, IRCCS, Rome, Italy; ^6^Institute of Biosciences and Bioresources, National Research Council, Naples, Italy; ^7^Department of Environmental, Biological and Pharmaceutical Science and Technologies, Università degli Studi della Campania “Luigi Vanvitelli”, Caserta, Italy; ^8^Center for Life Nano Science, Istituto Italiano di Tecnologia, Rome, Italy; ^9^D-Tails s.r.l., Rome, Italy; ^10^Core Facilities, Istituto Superiore di Sanità, Rome, Italy

**Keywords:** *CLTC*, clathrin, synaptic vesicles, motor behavior, *Caenorhabditis elegans*

## Abstract

*De novo CLTC* mutations underlie a spectrum of early-onset neurodevelopmental phenotypes having developmental delay/intellectual disability (ID), epilepsy, and movement disorders (MD) as major clinical features. *CLTC* encodes the widely expressed heavy polypeptide of clathrin, a major component of the coated vesicles mediating endocytosis, intracellular trafficking, and synaptic vesicle recycling. The underlying pathogenic mechanism is largely unknown. Here, we assessed the functional impact of the recurrent c.2669C > T (p.P890L) substitution, which is associated with a relatively mild ID/MD phenotype. Primary fibroblasts endogenously expressing the mutated protein show reduced transferrin uptake compared to fibroblast lines obtained from three unrelated healthy donors, suggesting defective clathrin-mediated endocytosis. *In vitro* studies also reveal a block in cell cycle transition from G0/G1 to the S phase in patient’s cells compared to control cells. To demonstrate the causative role of the p.P890L substitution, the pathogenic missense change was introduced at the orthologous position of the *Caenorhabditis elegans* gene, *chc-1* (p.P892L), via CRISPR/Cas9. The resulting homozygous gene-edited strain displays resistance to aldicarb and hypersensitivity to PTZ, indicating defective release of acetylcholine and GABA by ventral cord motor neurons. Consistently, mutant animals show synaptic vesicle depletion at the sublateral nerve cords, and slightly defective dopamine signaling, highlighting a generalized deficit in synaptic transmission. This defective release of neurotransmitters is associated with their secondary accumulation at the presynaptic membrane. Automated analysis of *C. elegans* locomotion indicates that *chc-1* mutants move slower than their isogenic controls and display defective synaptic plasticity. Phenotypic profiling of *chc-1* (+/P892L) heterozygous animals and transgenic overexpression experiments document a mild dominant-negative behavior for the mutant allele. Finally, a more severe phenotype resembling that of *chc-1* null mutants is observed in animals harboring the c.3146 T > C substitution (p.L1049P), homologs of the pathogenic c.3140 T > C (p.L1047P) change associated with a severe epileptic phenotype. Overall, our findings provide novel insights into disease mechanisms and genotype–phenotype correlations of *CLTC*-related disorders.

## 1. Introduction

*De novo* mutations in *CLTC* underlie a wide spectrum of infantile and childhood neurodevelopmental phenotypes, including developmental and epileptic encephalopathy (DEE), movement disorders (MD), and intellectual disability (ID) (DeMari et al., 2016; Lelieveld et al., 2016; Hamdan et al., 2017; Nabais Sá et al., 2020; Fernández-Mayoralas et al., 2021; Itai et al., 2022). The phenotypic spectrum is highly variable and ranges from mild ID with or without behavioral issues, particularly autistic features and attention deficit hyperactivity disorder (ADHD), to severe ID and refractory epilepsy. Most affected individuals present with global developmental delay (DD) and prominent hypotonia. Epilepsy is pharmacologically controlled only in a fraction of patients. Other features include nonspecific facial dysmorphisms, neuropsychiatric issues, and brain abnormalities, typically microcephaly and hypoplasia of the *corpus callosum*. Feeding difficulties, strabismus, and kidney, respiratory and hematological findings have been reported in a small fraction of affected subjects.

Although dominantly acting missense changes and small in-frame deletions seem to be more commonly associated with severe phenotypes compared with frank loss-of-function (LOF) variants (DeMari et al., 2016; Hamdan et al., 2017; Nabais Sá et al., 2020; Itai et al., 2022), genotype–phenotype correlations and the underlying pathogenic mechanisms remain largely uncharacterized. Of note, a narrow spectrum of missense changes have been associated with DD/ID and MD with no signs of epilepsy or callosal hypoplasia. Among these, the recurrent c.2669C > T (p.P890L; NM_004859.4) substitution has been reported in four unrelated individuals sharing a relatively mild neurodevelopmental phenotype (Hamdan et al., 2017; Manti et al., 2019; Nabais Sá et al., 2020). At the opposite side of the phenotypic spectrum associated with *CLTC de novo* variants, the c.3140 T > C missense change (p.L1047P) was reported in a single subject with severe ID and hypotonia, neonatal-onset epilepsy, and brain structural abnormalities (Hamdan et al., 2017).

*CLTC* encodes the widely expressed heavy chain of clathrin, a self-assembling vesicle coat protein involved in fundamental biological processes, including chromosome alignment on the mitotic spindle, endocytosis, intracellular trafficking, and synaptic vesicle (SV) recycling and regeneration (Royle et al., 2005; Kasprowicz et al., 2008; Fu et al., 2011; Robinson, 2015). Structurally, clathrin is composed of three heavy chains and three light chains, which assemble to form a “triskelion” (Fotin et al., 2004, 2006). In a triskelion, the heavy chains interact with each other in the central hub and protrude outwards to cooperate with other triskelia to form repeating hexagonal and pentagonal units, which, in turn, constitute the backbone of the clathrin coat. The trimeric structures assemble into lattices of different shape and size. Clathrin-coated vesicles are required for the delivery of a multitude of cargos from the plasma membrane and trans-Golgi network to their final destination (Kaksonen and Roux, 2018). A major process mediated by clathrin is the maintenance of SV pools at the presynaptic membranes through vesicle recycling, which enables the preservation of neurotransmission (Maycox et al., 1992; Cremona and De Camilli, 1997). This process takes place next to the active zone, the presynaptic region where exocytosis occurs, and is very similar to clathrin-mediated endocytosis occurring in non-neuronal cells. Finally, clathrin acts at the endosome compartment in the formation of new SVs (Murthy and De Camilli, 2003; Milosevic, 2018).

Here, we assessed the functional impact of the recurrent p.P890L substitution using complementary *in vitro* and *in vivo* experimental models. Specifically, patient-derived fibroblasts showed defective clathrin-mediated endocytosis and intracellular trafficking compared to three independent cell lines obtained from unrelated healthy donors, as well as the blockage of cell cycle transition from G0/G1 to the S phase as associated endophenotype. A gene-edited *Caenorhabditis elegans* strain carrying the p.P892L amino acid substitution in CHC-1/CLTC, corresponding to the pathogenic p.P890L change, was used to explore the consequences of the disease-causing variant *in vivo* and demonstrate its causative role in the identified phenotypes. Specifically, we document that the mutation acts dominantly causing defective release of neurotransmitters by different classes of neurons and their concomitant accumulation at the presynaptic membrane. Behavioral assessment reveals that mutant animals display aberrant locomotion and impaired synaptic plasticity. Finally, gene-edited worms harboring a second amino acid substitution (p.L1049P), which is homologous to the p.L1047P change causing a severe form of DEE, show an extremely severe phenotype due to an almost complete dominant-negative (DN) behavior of the mutant allele, providing first clues to the genotype–phenotype correlations observed in *CLTC*-related disorders.

## 2. Results

### 2.1. Patient-derived fibroblasts endogenously expressing CLTC^P890L^ show defective clathrin function and intracellular trafficking

**Figure 1:**
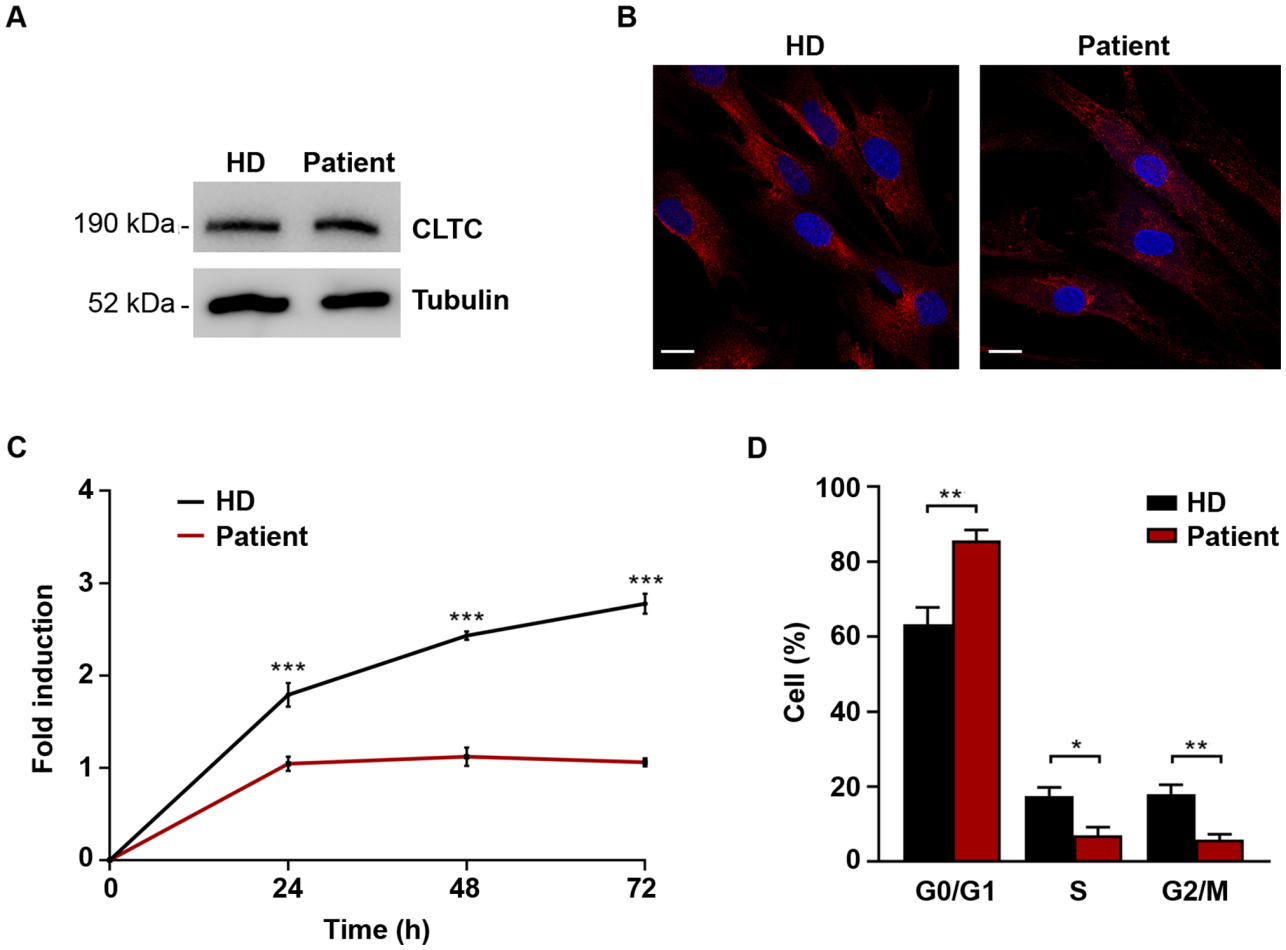
Cell cycle progression and cell proliferation are affected in primary fibroblasts carrying the *de novo* c.2669C > T (p.P890L) substitution in *CLTC*. **(A)** Western blot analysis on skin fibroblasts from affected and unaffected individuals shows no difference in CLTC levels. Blots were probed for β-tubulin as a loading control. Representative images of three independent experiments are shown. **(B)** Confocal microscopy analysis highlights a different distribution of CLTC *puncta* in primary fibroblasts from affected and unaffected individuals. Cells were stained with anti-CLTC antibody (red); nuclei were stained with DAPI (blue). Scale bar, 20 μm. **(C)** Proliferation is strongly reduced in cells harboring the pathogenic variant. Each point represents mean from triplicate plates. Values are normalized to time 0 and reported as fold changes, and results are expressed as mean values ± SEM. **(D)** Aberrant cell cycle profile determined by PI staining and FACS analysis is observed in cells from the affected individual. About 10,000 cells were analyzed in each experiment. Data represent mean ± SEM of three independent experiments. Statistical differences were obtained by two-way Anova followed by Sidak’s *post-hoc* test **(C)** and Student’s *t*-test **(D)** (*p < 0.05, **p < 0.01, ***p < 0.0001). HD, healthy donor.

**Figure 2:**
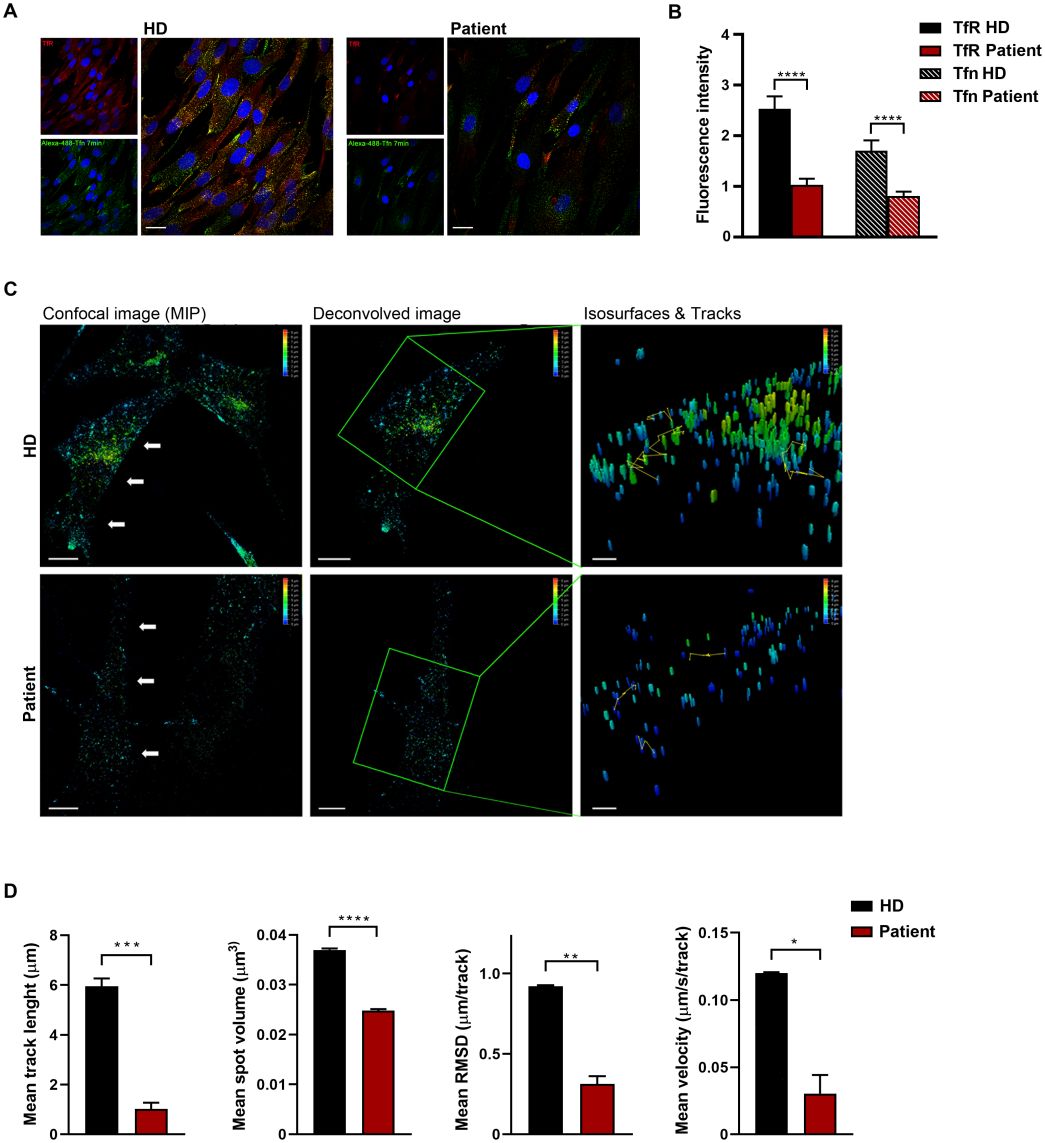
Defective clathrin-mediated endocytosis and intracellular trafficking in patient-derived fibroblasts. **(A)** Confocal microscopy analysis shows aberrant uptake of transferrin (Tf) in patient’s cells. Fibroblasts were serum starved, incubated with Alexa Fluor 488-conjugated Tf, and stained with anti-Tf receptor (TfR) antibody and Alexa Fluor 594 goat anti-mouse secondary antibody (red). Scale bar, 20 μm. **(B)** Quantification of total fluorescent intensity of TfR and Tfn (n = 66) highlights a significant decrease of fluorescent intensity of both the receptor and its ligand in patient’s fibroblasts compared to control cells (mean ± SEM from three independent primary cell lines). **(C)** Time-lapse imaging reveals that both the generation and transport of clathrin-coated vesicles are affected in cells obtained from the affected subject. Fibroblasts were incubated with Alexa 488-Tf and imaging was performed by XYZ stack acquisition. 3D projections are shown. Scale bar, 20 μm. The speed and traces of moving objects (yellow line) were analyzed using the LASX 3D software. Different colors of the particles correspond to different distribution of the vesicle (depth color coding scale on the top right, from 0 to 10 μm). **(D)** Morphometric 4D analysis shows reduced volume, velocity, length, and root mean square fluctuations of particles in patient-derived fibroblasts compared to control cells. For each biological replicate, five cells were measured. Data represent mean ± SEM of three independent experiments. Statistical differences were obtained using Student’s *t*-test with Bonferroni correction (*p < 0.05, **p < 0.01, ***p < 0.001, ****p < 0.0001). HD, healthy donor.

Codon 890 represents a mutational hotspot in *CLTC*. Specifically, the c.2669C > T (p.P890L) substitution has been identified in four unrelated subjects with a relatively mild DD/ID and MD phenotype (Hamdan et al., 2017; Manti et al., 2019; Nabais Sá et al., 2020). To explore the functional impact of this change, we first investigated the levels of the mutant protein and its subcellular localization in primary fibroblasts obtained from an affected individual heterozygous for the c.2669C > T change in *CLTC*. In these fibroblasts, exome sequencing data analysis excluded the occurrence of other functionally relevant variants compatible with known Mendelian disorders based on the expected inheritance model and clinical presentation (Supplementary Table S1). Immunoblotting analysis did not show any significant difference in the overall levels of wild-type (WT) and mutant protein (Figure 1A). By contrast, confocal microscopy observations indicated that CLTC^P890L^ shows a more scattered distribution compared to the WT counterpart (Figure 1B), suggesting an impaired ability of the mutant protein to form functional vesicles. By flow cytometry analysis, we observed a block in cell cycle progression from G0/G1 to the S phase, causing a significant slowdown of cell proliferation in patient’s fibroblasts compared to control cells (Figures 1C,D). Since cell cycle progression is strictly dependent on the binding of mitogenic factors to their cognate receptors, which, in turn, must be internalized to activate downstream signaling cascades (Olszewski et al., 2014), these findings suggested a defective intake of growth factors in cells harboring the pathogenic variant, resulting in cell growth drop-off. To test this hypothesis experimentally, a transferrin uptake assay was performed. Transferrin (Tf) is an iron-binding protein that facilitates iron-uptake into cells. Iron-loaded Tf (holo-Tf) binds to the Tf receptor (TfR) and is internalized via clathrin-mediated endocytosis (Pearse ad Robinson, 1990). Following incubation with fluorescent Tf, a reduced amount of Tf-positive vesicles and internalized TfR was observed in patient-derived cells compared to three independent primary fibroblast lines obtained from unrelated healthy individuals, which shared a homogeneous endophenotypic readout (Figures 2A,B and Supplementary Figure S1). This finding indicates that patient’s fibroblasts are partially unable to uptake Tf, suggesting a detrimental effect of the pathogenic p.P890L substitution on clathrin-mediated endocytosis and intracellular trafficking. Live imaging confirmed this finding, showing that both the volume and total amount of Tf-positive vesicles decreased in patient’s fibroblasts compared to control cells (Figure 2C and Supplementary Movie S1). We then analyzed the pattern of movements of Tf-positive vesicles as projection of 3D time-frames within the same cell. The tracking analysis of fluorescent vesicles indicated that the overall distance traveled by Tf-positive vesicles was significantly reduced in patient’s fibroblasts compared to control cells (Figure 2C). The depth color coding visualization of Tf-positive vesicles in mutant cells pointed out also a different axial distribution in patient-derived cells with a more concentrated signal near the plasma membrane (blue and light blue vesicles) compared to controls (from blue to yellow vesicles) (Figure 2C). Finally, morphometric 4D analysis showed that the volume of particles, as well as their velocity, length, and root mean square fluctuations (i.e., displacement of trajectories compared with a reference position over time) were dramatically reduced in fibroblasts expressing CLTC^P890L^ (Figure 2D).

In sum, *in vitro* studies pointed to defective clathrin-mediated endocytosis as a major altered process in cells expressing the p.P890L substitution in CLTC, which, in turn, was associated with flawed intracellular trafficking and slowdown in cell proliferation. While the causal role of the *CLTC* missense change is supported by its *de novo* origin in absence of any other clinically relevant variants in these cells (Supplementary Table S1), the contribution of other co-occurring event(s) to the observed altered processes in patient’s fibroblasts could not be ruled out *a priori*. The high mortality and extremely low transfection efficiency observed in rescue experiments designed to transiently overexpress the WT protein in these primary cells could not allow to collect consistent and reliable data. To overcome this issue, we turned to *C. elegans* as model system.

### 2.2. Generation and phenotypic characterization of a *Caenorhabditis elegans* model of the disease

Clathrin is highly conserved throughout evolution. *Chc-1*, the *C. elegans* ortholog of *CLTC*, displays 85% homology with the human gene, with 72% identity in amino acid sequence (Supplementary Figure S2; accessed January 15, 2023).^1^ Similar to its mammalian counterpart, *chc-1* is highly expressed in the nervous system (Sato et al., 2009). Knocking-down this gene via RNAi experiments (Grant and Hirsh, 1999; Chen et al., 2013; Shen et al., 2013) and functional assessment of a temperature sensitive null allele (Sato et al., 2009; Yu et al., 2018) indicated that the encoded protein is essential for endocytosis, SV recycling, and the generation of new SVs at the endosome compartment. When shifted to the restrictive temperature (25°C), null mutants present with defective yolk uptake, impaired locomotion and pharyngeal pumping, and arrested development (Sato et al., 2009). Knockout animals also display resistance to aldicarb-induced paralysis, suggesting a presynaptic defect in acetylcholine release at the level of the *C. elegans* neuromuscular junction (NMJ).

We introduced the c.2669C > T missense change at the orthologous position of the *C. elegans* gene via CRISPR/Cas9 to generate *chc-1*(*pan1*[P892L]) animals (hereafter *chc-1*[P892L]) (Supplementary Figure S3). The resulting nematodes, homozygous for the desired variant, did not show any obvious phenotype regardless of the breeding temperature, indicating that *chc-1*[P892L] does not behave as a severe LOF allele. In line with these findings, mutant oocytes were shown to efficiently internalize the YP170-GFP-tagged yolk protein (Supplementary Figure S4), suggesting the absence of any major effect on yolk endocytosis. Unfortunately, in our hands the null *chc-1*(*b1025*) temperature-sensitive allele generated by Sato and colleagues (Sato et al., 2009) displayed high penetrant embryonic lethality and larval arrest, even when animals were grown at the semi-permissive (20°C) or permissive (15°C) temperatures, making it impossible to use this strain as a positive control in our experiments.

### 2.3. Effect of the *chc-1*[P892L] allele on synaptic transmission

Based on clathrin’s pivotal role in the neoformation of SVs at the endosome compartment and in mediating vesicle recycling, we hypothesized that *chc-1*[P892L] mutants might show a generalized deficit in synaptic transmission rather than a defect involving one or more specific neurotransmitters. To test this hypothesis, we performed a panel of pharmacological and behavioral assays aimed at exploring the impact of the CHC-1 p.P892L substitution on cholinergic, GABAergic, and dopaminergic signaling.

**Figure 3:**
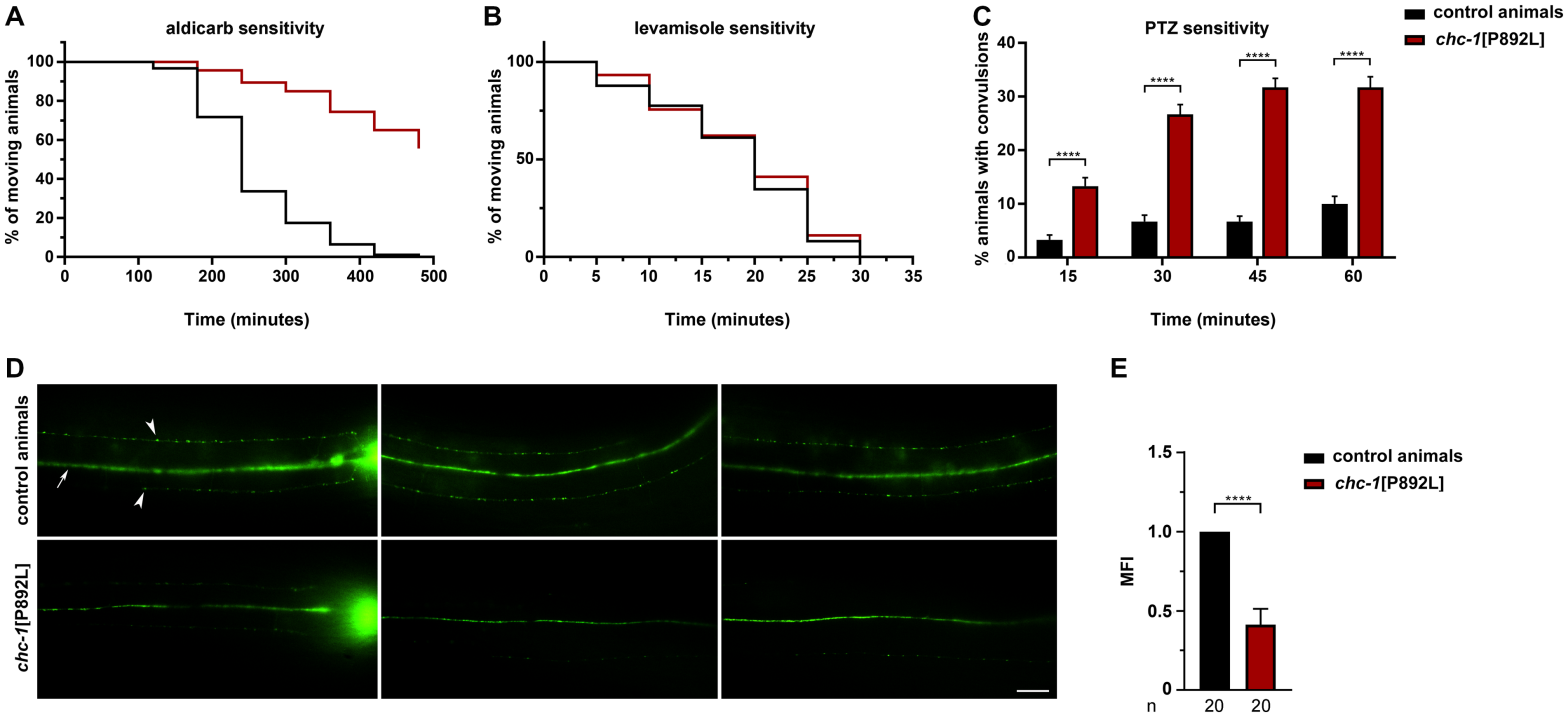
*chc-1*[P892L] animals show reduced neurotransmitters’ release at the *C. elegans* NMJ. **(A)** *chc-1* mutants display partial resistance to aldicarb-induced paralysis (500 μM) compared with control animals (*p* < 0.0001; log-rank test), suggesting decreased ACh release by ventral cord motor neurons. Control animals, *n* = 90; *chc-1*[P892L], *n* = 150. **(B)** *chc-1*[P892L] animals show normal sensitivity to the paralyzing effect of the cholinergic agonist levamisole (1 mM), supporting the presynaptic origin of the defect. Control animals, *n* = 50; *chc-1*[P892L], *n* = 95. **(C)** *chc-1* mutants display hypersensitivity to PTZ-induced anterior convulsions (5 mg/mL on agar plates), likely indicating defective GABA release (****p < 0.0001 in all comparisons; Fisher’s exact test with Bonferroni correction). Control animals, *n* = 30; *chc-1*[P892L], *n* = 60. Data represent mean ± SEM of at least three independent experiments **(A–C)**. **(D)** Dorsal view shows the dorsal nerve cord (arrow) and small fluorescent *puncta* (arrowheads) at the sublateral nerve cords. The nerve ring is also visible (left panels). *chc-1*[P892L] animals show a significant reduction in the number and/or fluorescent intensity of *puncta*. Twenty animals for each genotype were analyzed. **(E)** The Mean Fluorescence Intensity (MFI) was quantified using the NIH Image J software (****p < 0.0001; unpaired *t*-test with Welch’s correction).

We first measured sensitivity of gene-edited animals to aldicarb, an inhibitor of acetylcholinesterase, which causes accumulation of acetylcholine in the synaptic cleft, leading to sustained muscle contraction and paralysis (Rand, 2007). Aldicarb toxicity can be ameliorated by mutations that reduce neurotransmitter release at the NMJ. Of note, homozygous *chc-1*[P892L] animals behaved as null mutants (Sato et al., 2009), displaying partial resistance to aldicarb-induced paralysis (Figure 3A). This finding suggests defective release and/or recycling of acetylcholine by excitatory ventral cord motor neurons. Since animals can be less sensitive to aldicarb also because of ineffective postsynaptic response to acetylcholine, which may be due to a decreased number or defective function of levamisole-sensitive receptors, we measured sensitivity to levamisole, a nicotinic receptor agonist acting on postsynaptic muscles (Rand, 2007). No significant difference in sensitivity to this drug between WT and *chc-1*[P892L] mutants was observed (Figure 3B), further supporting the presynaptic origin of the defect. Finally, aldicarb responsiveness is modulated by the balance of excitatory (cholinergic) and inhibitory (GABAergic) inputs onto muscles (Loria et al., 2004; Jiang et al., 2005; Vashlishan et al., 2008). We then assessed GABAergic signaling by measuring sensitivity to pentylenetetrazole (PTZ), a competitive antagonist of GABA_A_ receptors expressed on body-wall muscles and head muscles. PTZ assay provides a fast and reliable technique to reveal presynaptic defects in GABA release (Thapliyal and Babu, 2018). Exposure to this drug leads to a shift in the equilibrium between excitatory and inhibitory signals toward the former, resulting in a PTZ-induced anterior convulsion phenotype. Notably, *chc-1*[P892L] animals showed strong hypersensitivity to PTZ (Figure 3C), indicating flawed GABAergic transmission. In line with these findings, the SV pool at the ventral sublateral nerve cords was affected in *chc-1*[P892L] animals expressing GFP-tagged synaptogyrin (SNG-1), which localizes to SVs in *C. elegans* neurons (Zhao and Nonet, 2001; Figures 3D,E).

Based on the clinical findings linking the p.P890L variant to MD, dopaminergic signaling was also evaluated. In *C. elegans*, dopamine plays a key role in the response to environmental changes by modulating the locomotor behavior (Sawin et al., 2000). Typically, nematodes slow down when they find a bacterial lawn to increase the amount of time spent in the presence of food. This behavior, which is known as “basal slowing response” (BSR), is mediated by dopamine. As expected (Lints and Emmons, 1999), animals harboring a LOF allele of *cat-2*, encoding the tyrosine hydroxylase enzyme that converts tyrosine to levodopa, the biosynthetic precursor of dopamine, did not show any BSR response (Supplementary Figure S5A). Conversely, *chc-1*[P892L] nematodes behaved as control animals in this assay. To reveal possible occurrence of a more subtle defect in dopaminergic signaling, a more sensitive assay (i.e., swimming induced paralysis, SWIP) was carried out in a sensitized genetic background. While control animals swim vigorously when placed in a liquid solution, worms harboring a LOF mutation in the gene coding for DAT-1, the presynaptic transporter involved in dopamine reuptake, are characterized by a rapidly-onset SWIP phenotype, which was shown to be caused by extrasynaptic accumulation of dopamine (McDonald et al., 2007). Following 10 min spent in M9 solution, 74% of *dat-1*(*ok157*) knockout animals were paralyzed, compared to 16 and 12% of WT and *chc-1*[P892L] worms, respectively (Supplementary Figure S5B). More importantly, the *chc-1* mutant allele ameliorated the SWIP phenotype of *dat-1* mutants (55% of paralyzed animals), suggesting that p.P892L also affects, albeit slightly, dopamine release. A postsynaptic effect of the mutation on the activation of dopamine D2-like receptors, however, cannot be ruled out (Carvelli et al., 2010; Kudumala et al., 2019).

Overall, combined results from drug-sensitivity and behavioral assays revealed that *chc-1*[P892L] mutants show a generalized defect in synaptic transmission.

**Figure 4:**
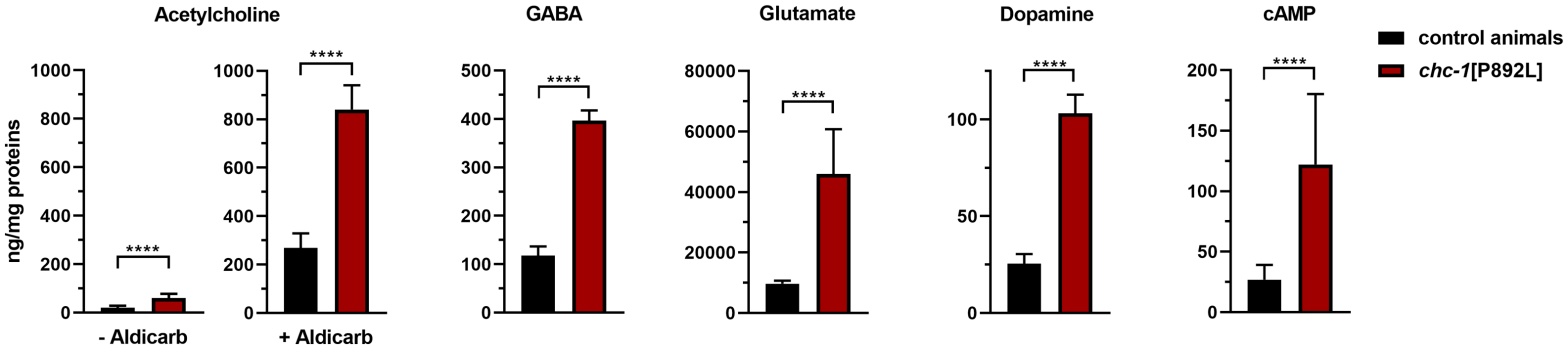
UHPLC–MS/MS analysis shows an overall increase in the total amount of neurotransmitters in *chc-1*[P892L] animals. Ultra-performance liquid chromatography–tandem mass spectrometry (UHPLC–MS/MS) analysis was performed using protein lysates obtained from ~5,000 L3 larvae. A significant increase in the total amount of acetylcholine, GABA, glutamate, dopamine, and cyclic AMP (cAMP) is observed in *chc-1*[P892L] animals, suggesting that defective release of synaptic vesicles is coupled to the accumulation of neurotransmitters at the presynaptic membrane (****p < 0.0001 in all comparisons; unpaired *t*-test with Welch’s correction). Data represent mean ± SEM of three independent experiments.

Defective formation/release of SVs could lead to the accumulation of neurotransmitters at the presynaptic terminal. To test this hypothesis, ultra-performance liquid chromatography–tandem mass spectrometry (UHPLC–MS/MS) analysis was performed on ~5,000 L3 larvae to quantify the levels of glutamate, acetylcholine, GABA, and dopamine in *chc-1*[P892L] animals compared to control worms. The analysis documented an overall increase in the total amount of neurotransmitters in gene-edited worms, suggesting that defective release of SVs due to aberrant clathrin function likely leads to the accumulation of neurotransmitters at the presynaptic membrane (Figure 4 and Supplementary Figure S6). Similarly, cyclic AMP (cAMP) levels were found to be doubled in mutant animals compared to isogenic controls. Based on the modulatory role of the cAMP-PKA signaling cascade on the secretion of SVs via phosphorylation of synaptic targets (Gekel and Neher, 2008; Wang and Sieburth, 2013), this finding suggests the presence of a compensatory mechanism aimed at boosting the function of defective SV release machinery in *chc-1*[P892L] mutants.

Taken together, the collected data demonstrated that p.P892L negatively impacts synaptic transmission by reducing SV release/recycling by different classes of *C. elegans* neurons.

### 2.4. *chc-1*[P892L] animals display aberrant locomotion and flawed synaptic plasticity

Locomotion and learning assays were carried out to assess the consequences of the mutation on *C. elegans* behavior and to identify relevant phenotypes for future genetic or chemical screening.

**Figure 5:**
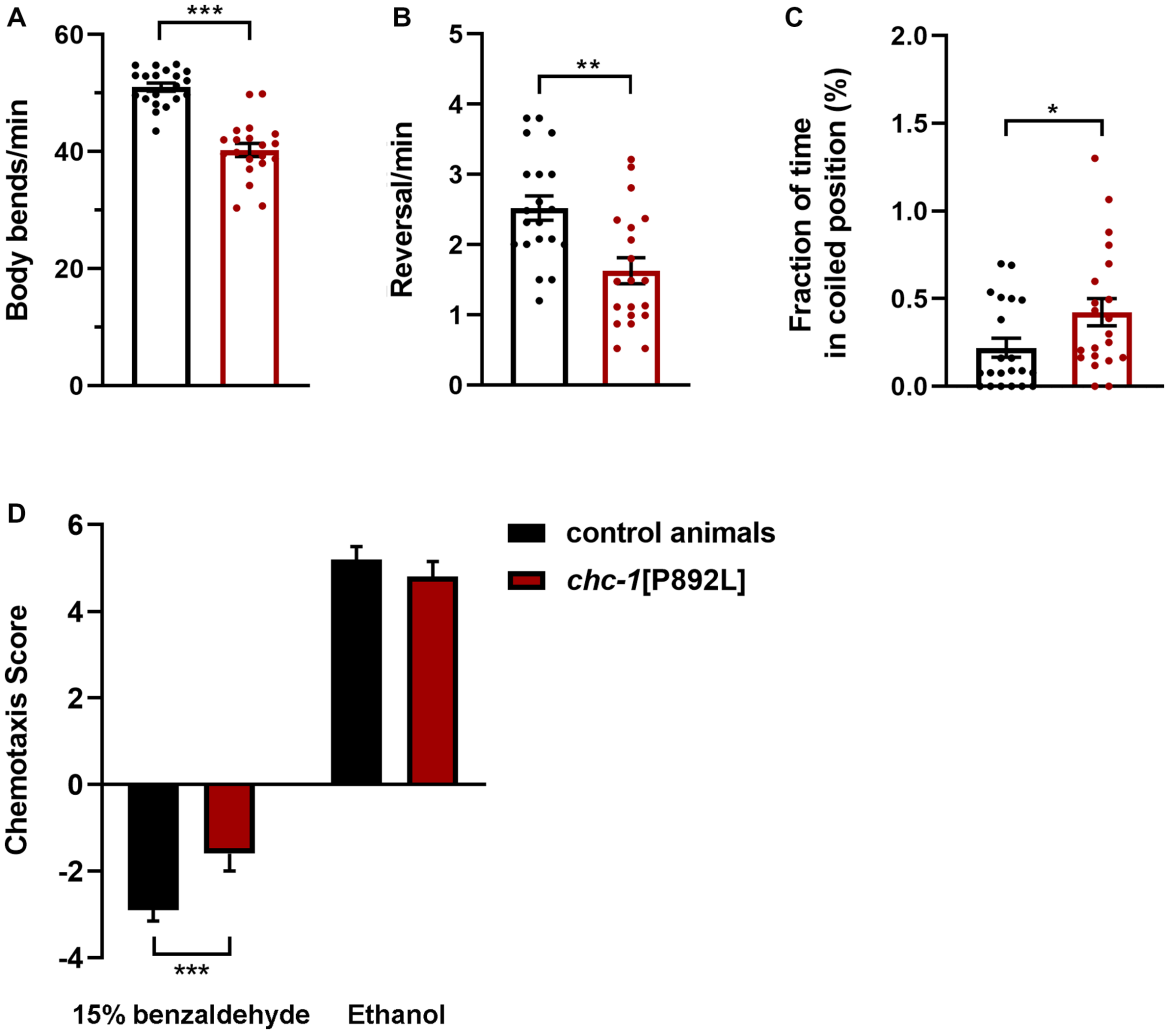
*chc-1*[P892L] animals exhibit aberrant locomotion and defective synaptic plasticity. The aberrant locomotor activity of *chc-1* mutants is revealed by the lower number of body bends **(A)** and reversals **(B)** per minute compared with control animals, and the average fraction of time spent in a coiled position **(C)** (*p < 0.05, **p < 0.002, ***p < 0.0001; unpaired *t*-test with Welch’s correction). Twenty animals for each genotype were assayed. Data represent mean ± SEM of multiple observations. **(D)** Single-worm chemotaxis assays were performed in the presence of 1% benzaldehyde following conditioning with 15% benzaldehyde in the absence of food. A positive and negative chemotaxis index indicate attraction and aversion to the odorant, respectively. While control animals conditioned with ethanol are attracted to benzaldehyde, those conditioned with benzaldehyde avoid this odorant because they associate it with the absence of food. Conditioned *chc-1*[P892L] mutants avoid benzaldehyde less efficiently compared to control worms, indicating reduced learning or memory abilities (***p < 0.0001; unpaired *t*-test with Welch’s correction). Twenty animals for each genotype were tested. Data represent mean ± SEM.

*chc-1* deficiency has been reported to reduce the thrashing activity of worms cultured at the permissive temperature, resulting in almost completely paralyzed animals when shifted to the restrictive temperature (Sato et al., 2009). To characterize the locomotor behavior of *chc-1*[P892L] animals, we used a recently developed automated tracking platform (Di Rocco et al., 2022). Computational analysis revealed that *chc-1*[P892L] animals are characterized by less frequent body bends compared to control worms, indicating hypoactive crawling (Figure 5A). Mutant nematodes also exhibited a significant reduction in the reversal (i.e., change of direction) rate (Figure 5B), which is likely due to weakened release of glutamate onto AVA, the main interneuron governing this repetitive behavior (Katz et al., 2019). Finally, *chc-1*[P892L] mutants showed uncoordinated locomotion, assuming a coil-like shape when attempting to move (“coiler” phenotype) and maintaining this position for longer than control animals (Figure 5C).

*C. elegans* exhibit a remarkable ability to learn and remember environmental conditions predicting the presence of adverse stimuli, which enables the animals to chemotax, aerotax or thermotax toward more favorable environments (Ardiel and Rankin, 2010). To explore whether aberrant synaptic transmission caused by impaired clathrin function may affect cognition, we challenged animals in a learning assay that was performed following conditioning with high doses of benzaldehyde. Synchronized adult hermaphrodites were conditioned with 15% benzaldehyde in plates without food and then tested against 1% benzaldehyde in single-worm chemotaxis assays. After conditioning, control animals associated the odorant with unfavorable conditions (i.e., absence of food). Therefore, they were less attracted to benzaldehyde compared to mock animals that have been conditioned with neutral ethanol (Figure 5D). Differently, conditioned *chc-1*[P892L] mutants were shown to less efficiently avoid benzaldehyde compared to control animals, indicating defective learning and/or memory.

### 2.5. The p.P890L variant has a mild dominant-negative effect in *Caenorhabditis elegans* neurons

**Figure 6:**
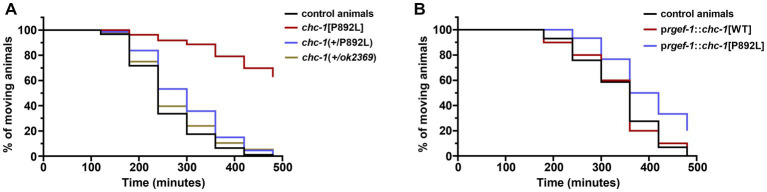
Multiple genetic approaches suggest that p.P892L substitution functions as a mild dominant-negative allele in *C. elegans* neurons. **(A)** Sensitivity to aldicarb-induced paralysis was assessed in worms carrying the *chc-1* variant in a heterozygous state. While *chc-1* haploinsufficient animals—*chc-1(+/ok2369)*—show normal sensitivity to aldicarb, *chc-1*(+/P892L) hermaphrodites paralyze slightly slower than control worms (*p* = 0.05; log-rank test). Thirty animals for each genotype were tested. Data represent mean ± SEM of multiple experiments performed using four independent clones generated by genetic crosses to control animals. **(B)** Aldicarb sensitivity was also assessed in transgenic animals overexpressing the WT or mutant form of *chc-1* pan-neuronally. Again, we observed a mild but significant resistance to aldicarb following overexpression of the mutant but not the WT *chc-1* allele (*p* < 0.005; log-rank test). Thirty animals for each genotype were tested. Data represent mean ± SEM of multiple experiments performed using three transgenic lines for each genotype.

In a clinical setting, the pathogenic *CLTC* variants are dominant, with WT and mutant clathrin molecules being expressed simultaneously. To further model the genetic change occurring in *CLTC*, we analyzed the F1 progeny after crossing homozygous *chc-1*[P892L] hermaphrodites to control males carrying the WT *chc-1* allele, and assessed whether this was sufficient to cause a phenotype. *Chc-1*(+/*ok2369*) heterozygotes showed no aldicarb hypersensitivity (Figure 6A) or locomotion defects. This finding suggested that, unlike human *CLTC*, which is intolerant to monoallelic LOF variants (pLI = 1, accessed January 15, 2023),^2^ *chc-1* does not cause haploinsufficiency, at least for the analyzed phenotypes. In contrast, *chc-1*(+/P892L) hermaphrodites showed mild resistance to aldicarb-induced paralysis, though less evident compared to what had been observed in homozygous *chc-1*[P892L] mutants, suggesting that p.P892L acts dominantly by interfering with the function of the WT protein. To further test the DN activity of the mutant form of clathrin, we performed transgenic overexpression studies using the *rgef-1* pan-neuronal promoter to overexpress *chc-1*[P892L] in all neurons of WT animals. Again, we observed a slight but significant resistance to aldicarb following overexpression of the mutant but not of the WT *chc-1* allele (Figure 6B), confirming the mild DN behavior of the mutant allele.

### 2.6. Neuronal expression of wild-type *chc-1* improves the phenotype of *chc-1*[P892L] mutants

**Figure 7:**
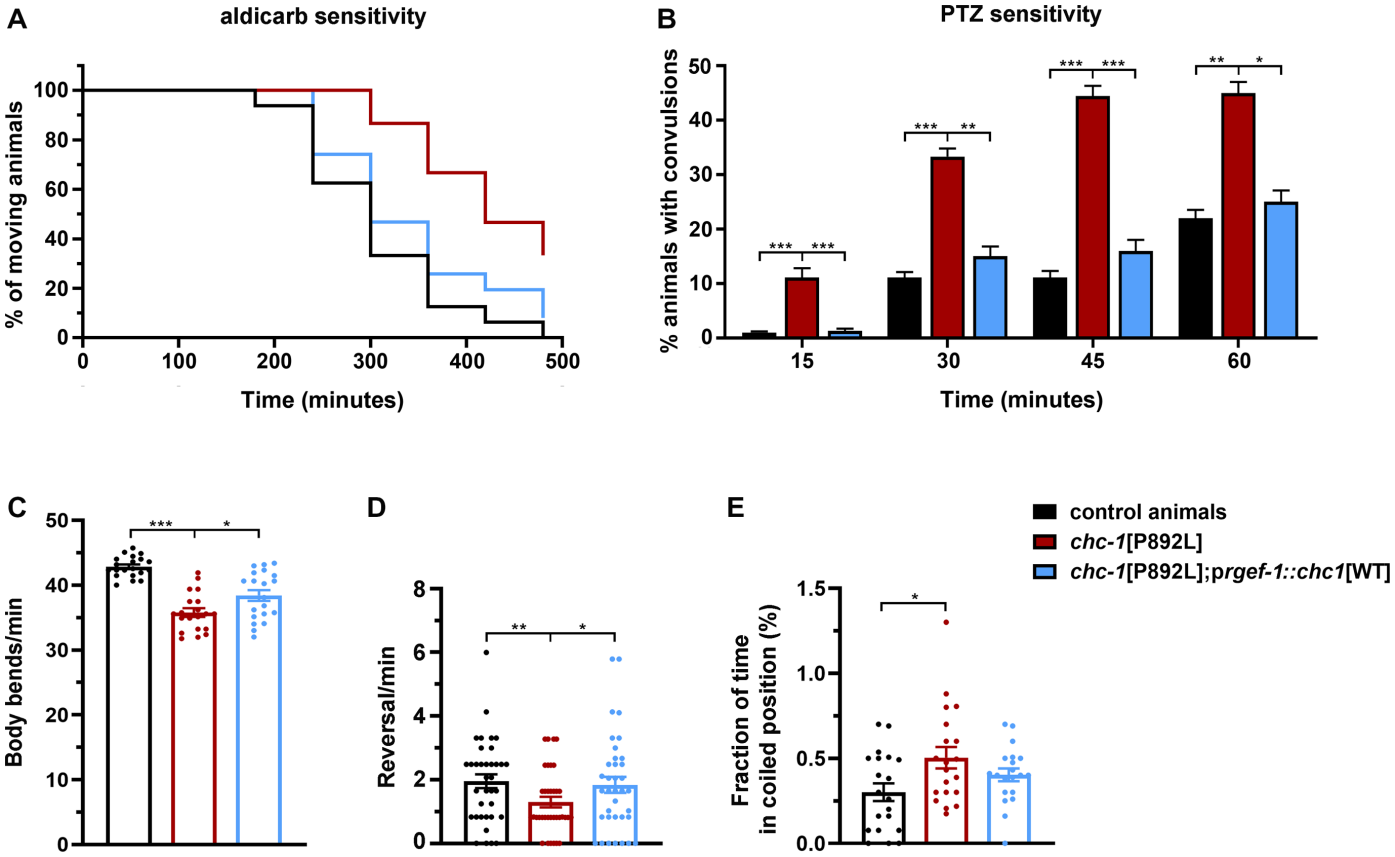
Neuronal expression of wild-type *chc-1* improves the phenotype of *chc-1*[P892L] mutants. Normal sensitivity to aldicarb (500 μM; *p* < 0.0001, log-rank test) **(A)** and PTZ (5 mg/mL; *p < 0.05, **p < 0.01***p < 0.001, Fisher’s exact test with Bonferroni correction) **(B)** is restored in *chc-1*[P892L] animals following overexpression of the WT *chc-1* allele in neurons driven by the *rgef-1* promoter. Control animals, *n* = 50 and *n* = 30; *chc-1*[P892L], *n* = 50 and *n* = 30; *chc-1*[P892L]; p*rgef-1::chc-1*[WT] 2 ng, *n* = 60 and *n* = 30 for aldicarb and PTZ assays, respectively. Data represent mean ± SEM of at least three independent experiments. Neuronal expression of WT *chc-1* ameliorates aberrant locomotion in terms of body bend per minute **(C)**, reversal per minute **(D)**, and the average fraction of time spent in a coiled position (albeit not significantly) **(E)** (*p < 0.05, **p < 0.01, ***p < 0.001; One-Way Anova with Tukey’s multiple comparisons test). Twenty animals for each genotype were assayed. Data represent mean ± SEM of multiple observations.

Although control and gene-edited nematodes are expected to be isogenic, we generated transgenic lines overexpressing the WT form of CHC-1 in neurons of *chc-1*[P892L] animals to further demonstrate the causative role of the p.P892L substitution in the identified phenotypes and rule out the presence of a residual off-target mutation in the genomic background of the mutant strain. Analysis of a subset of phenotypes revealed a clear, albeit incomplete, effect of the transgene in restoring normal locomotion and sensitivity to aldicarb and PTZ (Figure 7). Therefore, we conclude that aberrant neurotransmitters’ release observed in *chc-1*[P892L] mutants can be directly ascribed to the p.P892L substitution.

### 2.7. A severe developmental phenotype is associated with the p.L1047P variant causing DEE

On the opposite side of the phenotypic spectrum associated with *CLTC* mutations, a subset of missense changes causes DEE. Among these, the c.3140 T > C (p.L1047P) substitution was reported in a 16 year-old boy with severe ID, neonatal-onset hypotonia and epilepsy, brain structural abnormalities (i.e., thin and short *corpus callosum*), and acquired microcephaly (Hamdan et al., 2017). To explore the functional impact of this variant *in vivo*, we introduced the c.3146 T > C nucleotide substitution at the orthologous position of the *C. elegans* gene to generate *chc-1*[L1049P] animals. Following multiple rounds of injections, we failed in identifying worms carrying the desired change. Remarkably, we noted a number of unhatched eggs and few larvae arrested at the L1/L2 stage. Genotyping of death larvae disclosed the occurrence of the homozygous c.3146 T > C nucleotide substitution (Supplementary Figure S3), indicating that this variant, when present in the homozygous state, is embryonic lethal or causes early larval arrest, at both 15 and 20°C. Moreover, a significant proportion of hermaphrodites (~30%) was sick and almost paralyzed, grew slowly, laid a small number of eggs, and died within a few days of adulthood. Genotyping of sick animals revealed the presence of the variant in a heterozygous state (Supplementary Figure S3), highlighting a strong DN behavior for the *chc-1*[L1049P] allele.

## 3. Discussion

A wide spectrum of heterozygous *CLTC* variants have been reported to cause a variable neurodevelopmental phenotype characterized by mild to severe ID, microcephaly, epilepsy, and brain structural abnormalities. Here, we characterized the functional behavior of the recurrent p.P890L *CLTC* variant that has been associated with relatively mild clinical features, using complementary *in vitro* and *in vivo* models. We show that primary fibroblasts heterozygous for the missense change show defective clathrin-mediated endocytosis and intracellular trafficking. When introduced in the *C. elegans* genome, this variant causes locomotor and learning defects due to defective release of multiple neurotransmitters and their secondary accumulation at the presynaptic membrane. We also provided data supporting the hypomorphic nature of the variant and its mild DN behavior. Of note, a more severe phenotype resulting from a strong DN effect was demonstrated for a second *CLTC* variant (p.L1047P), which mirrors the severe clinical phenotype reported in the affected subject. Overall, our findings provide a mechanistic explanation for the genotype–phenotype correlations reported in *CLTC*-related diseases.

Clathrin is part of a network of proteins regulating vesicular transport and endocytosis, and defects in clathrin assembly are expected to have a pleiotropic effect on multiple cellular processes. Clathrin-mediated endocytosis is a ubiquitous event operating in all cell types providing a pathway for internalization of extracellular signals and receptors. Defective function of clathrin-mediated vesicular transport has a dramatic impact in neurons since it is required for SV recycling at the presynaptic compartment and regulates proper trafficking of postsynaptic receptors, which is a key event controlling fine-tuning of signal strength during neurotransmission (Jung and Haucke, 2007). Consistently, aberrant vesicular transport and endocytosis have been involved in the pathophysiology of various neurodevelopmental and neurodegenerative disorders. Indeed, a number of genes coding for proteins mediating endocytosis and SV dynamics have been associated with early-onset Parkinson’s disease (PD) (Roosen et al., 2019), which is in line with the clinical features we had previously reported in an adult patient harboring the p.P890L substitution of CLTC (Manti et al., 2019). Among these genes, synaptojanin 1, encoded by *SYNJ1*, controls the detachment of accessory clathrin proteins from the vesicular membrane before coat disassembling (Cremona et al., 1999). Biallelic variants in this gene cause a recessive form of juvenile PD (PARK20, MIM #615530). Similarly, mutations in *DNAJC6*, encoding auxilin 1, a member of the DnaJ homolog C (DNAJC) heat shock protein family that modulates clathrin coat removal (Ahle and Ungewickell, 1990), underlie early-onset PD (PARK19A/B, MIM# 615528). Additional members of this family, including DNAJC12 and CSPα-DNAJC5, have been associated with non-progressive L-DOPA responsive parkinsonism (MIM #617384) and neuronal ceroid lipofuscinosis with generalized seizures/MD (MIM #162350), respectively (Benitez et al., 2011; Nosková et al., 2011; Straniero et al., 2017). Finally, pathogenic variants affecting other genes involved in endomembrane trafficking, such as *ATP13A2*, *LRRK2*, *SNCA*, and *VPS35*, have been associated with monogenic forms of PD (Hardy et al., 2009). Overall, these findings strongly support the importance of clathrin function for motor control and healthy neuronal behavior.

Thirty-one individuals with disease-causing *CLTC* variants have been reported so far (DeMari et al., 2016; Lelieveld et al., 2016; Hamdan et al., 2017; Nabais Sá et al., 2020; Fernández-Mayoralas et al., 2021; Itai et al., 2022). Affected subjects exhibit a wide variety of clinical features and variable severity of the phenotype, which is largely dependent on the mutation type and affected residue. Specifically, missense changes and in-frame deletions were reported to result in more severe phenotypes compared to LOF mutations (i.e., nonsense, frameshift, and splice-site changes) (Hamdan et al., 2017; Nabais Sá et al., 2020; Itai et al., 2022). It has been postulated that the former class of variants affects triskelion assembly, altering the overall structure of the coated vesicle through DN mechanisms. Conversely, LOF variants have been predicted to result in *CLTC* haploinsufficiency. Exception to this general rule is represented by the recurrent p.P890L substitution, which has been associated with global DD generally evolving into mild ID, with no epilepsy, in four unrelated individuals (Hamdan et al., 2017; Manti et al., 2019; Nabais Sá et al., 2020). Three subjects harboring this variant have been reported with mild ataxia and mild hypotonia, with oral and motor apraxia. MRI abnormalities were documented in one subject showing periventricular white matter alterations at 23 months of age. One adult patient, a 30 year-old woman, presented with early-onset DD and during adolescence experienced a mild cognitive decline and a parkinsonian phenotype (relapsing–remitting hypokinetic-rigid syndrome with severe achalasia, weight loss, and mood disorder) associated with low levels of neurotransmitters’ metabolites in CSF (Manti et al., 2019). A second exception to the proposed model is represented by a subset of nonsense and frameshift mutations that causes a severe DEE phenotype. While our understanding of the pathogenic mechanism(s) underlying the functional consequences of the latter class of changes requires dedicated effort, our data provide a rationale for the relatively mild effect of the p.P890L substitution.

Our work provide further evidence of the helpfulness of *C. elegans* as an experimental model to dissect the pathophysiology of neurological disorders (Kaletta and Hengartner, 2006). This system offers a unique opportunity to define the molecular mechanisms by which *CLTC* mutations affect synaptic transmission, and elucidate the basis underlying the observed genotype–phenotype correlations. *In vivo* data demonstrated that p.P890L and p.L1047P differentially affect embryonic development, endocytosis, and maintenance of the SV pool in neurons. On the one hand, p.P890L was shown to weaken synaptic transmission, at least at the cholinergic, GABAergic, and dopaminergic synapses, but had no major effect on yolk uptake and key developmental processes. A detrimental effect of this change on clathrin-mediated endocytosis was revealed in primary fibroblasts from an affected individual. On the other hand, p.L1047P severely affected embryonic and larval vitality. These findings support a model in which distinct thresholds for clathrin activity are required to induce neuronal- or developmental-specific phenotypes. Accordingly, patients harboring these variants show clinical features of diverse severities and a different age of onset.

In sum, we demonstrate that *CLTC* mutations cause a generalized deficit in synaptic transmission likely due to aberrant formation/turnover of SVs. Our findings also establish an informative *in vivo* experimental platform for future genetic and pharmacological screens aimed at the identification of new therapeutic strategies for *CLTC*-related disorders.

## 4. Materials and methods

### 4.1. Primary cell lines

Control and patient’s fibroblasts were isolated from dermal skin biopsies and were cultured in Dulbecco’s Modified Eagle’s Medium (DMEM) (Sigma-Aldrich) supplemented with 10% heat-inactivated FBS, 2 mM glutamine, 100 U/mL penicillin and 100 μg/mL streptomycin (Euroclone), and maintained at 37°C in a humidified atmosphere containing 5% CO_2_. Cells were collected according to procedures conforming to the ethical standards of the declaration of Helsinki protocols and approved by the review boards of the involved institutions (“Sapienza” University of Rome; Ospedale Pediatrico Bambino Gesù, Rome). Signed informed consent was obtained from each participating subject. Exome sequencing in patient-derived fibroblasts excluded the occurrence of additional functionally relevant variants compatible with known Mendelian disorders (Supplementary Table S1). Similarly, exome sequencing in the three control fibroblast lines excluded the occurrence of functionally relevant variants in known disease-causing genes.

### 4.2. *In vitro* studies

Primary fibroblasts were seeded at 25 × 10^4^ in 6-well plates and analyzed after 48 h. Western blot analyses were performed as previously described (Muto et al., 2018; Motta et al., 2019). Experiments used the following antibodies: rabbit monoclonal anti-CLTC (1:1,000, Cell Signaling), mouse monoclonal anti-β-tubulin (1:1,000, Sigma-Aldrich), and anti-mouse and anti-rabbit HRP-conjugated secondary antibodies (1:3,000, Sigma-Aldrich).

For FACS analysis, fibroblasts were seeded at 7 × 10^4^ in 6-well plates and assessed after 48 h. Cells were fixed in Acetone/Methanol (1:1) pre-cooled to −20°C, kept in the fixing solution for 1 h at 4°C, and then spun down for 2 min at 4,000 rpm. After washing with ice-cold PBS, pellets were resuspended in 0.5 mL PBS containing 10 μg/mL RNaseA and 20 μg/mL Propidium Iodide stock solution, transferred to FACS tubes, and incubated at RT in the dark for 30 min. FACS analysis was performed using a FACS-Calibur (BD Biosciences).

Cell proliferation was determined by a sulforhodamine B (SRB) colorimetric assay. Fibroblasts were seeded at 2 × 10^4^ in 24-well plates and assessed after 24, 48, and 72 h. Cells were fixed with cold 40% trichloroacetic acid (Sigma-Aldrich) and incubated at 4°C for 1 h. Plates were rinsed five times with cold water, allowed to dry at RT, and then stained with 0.4% SRB solution (Sigma-Aldrich) for 30 min. Cells were then washed with 1% acetic acid (Merck Millipore). Protein-bound precipitates were dissolved in 10 mM Tris (pH 10.5) (Merck Millipore). Plates were read at 492 nm using an Infinity 200 plate reader (Tecan).

For transferrin uptake, 2 × 10^4^ fibroblasts grown on coverslips were serum-starved for 45 min at 37°C. Cells were then incubated with 50 μg/mL Alexa 488-conjugated Tf (Invitrogen) in a serum-free medium for 7 min, washed twice on ice-cold PBS to remove unbound Tf, and then incubated twice for 2 min at 4°C in ice-cold stripping buffer (150 mM NaCl, 20 mM HEPES, 5 mM KCl, 1 mM CaCl_2_, 1 mM MgCl_2_, pH 5.5) to remove cell surface-bound Tf. Cells were then fixed with 3% paraformaldehyde for 30 min at 4°C and stained with mouse monoclonal anti-TfR antibody (1:250, Thermo Fisher) for 3 h at RT, rinsed twice with PBS, and incubated with Alexa Fluor 594 goat anti-mouse secondary antibody (1:200, Molecular Probes). To visualize protein subcellular localization, cells were stained with rabbit monoclonal anti-CLTC antibody (1:50, Cell Signaling), rinsed twice with PBS, and incubated with Alexa Fluor 594 goat anti-rabbit secondary antibody (1:200, Molecular Probes). Confocal microscopy was performed on a Leica TCS SP8X laser-scanning confocal microscope (Leica Microsystems) and sequential confocal images were acquired using HC PL APO CS2 63x.

Live imaging were carried out using 3 × 10^4^ cells seeded in 35 mm dishes (Ibidi) for 24 h, serum-starved for 45 min at 37°C, and incubated with 50 μg/mL Alexa 488-conjugated Tf (Invitrogen) in serum-free medium for 7 min. Live imaging of Tf uptake was conducted using a TCS-SP8X Leica confocal microscope equipped with a resonant scanner for fast imaging and a cell incubator. 3D time series, spaced 0.5 μm, were acquired for 5 min at 8.2-s intervals. Deconvolution analysis (HyVolution2 software, Leica Microsystems) was applied to z-stacks to improve contrast and resolution of raw images, which were imported into the tracking module of the LASX 3D software (Leica Microsystems) to get 3D images and measure the speed and tracking of moving objects. Objects tracks of each 4D time series were defined as described (Egorov et al., 2009; Blas-Rus et al., 2016).

### 4.3. *Caenorhabditis elegans* studies

Culture, maintenance, injections and genetic crosses were carried out using standard techniques (Sulston and Hodgkin, 1988; Mello et al., 1991). Control animals (Bristol N2) and the DH1230 *chc-1(b1025)*, VC2405 *chc-1(ok2369)*, RT130 *pwIs23 (vit-2::GFP)*, NM1233 *jsIs219 ((pSY3) sng-1::GFP + rol-6(su1006))*, RM2702 *dat-1(ok157)*, and CB1112 *cat-2(e1112)* strains were provided by the *Caenorhabditis Genetics Center* (CGC) (University of Minnesota, Minneapolis, MN).

Engineered changes to the endogenous *chc-1 locus* were performed by CRISPR/Cas9, as previously described (Motta et al., 2020; Di Rocco et al., 2022). Twenty (*chc-1*[P892L]) and sixty (*chc-1*[L1049P]) N2 animals have been injected with a mix containing 750 ng/μL Cas9 (IDT), 700 ng/μL ALT-R CRISPR tracrRNA, 115 ng/μL *dpy-10* crRNA, 37.5 ng/ μL ssODN *dpy-10*, 350 ng/μL *chc-1*[P892L] crRNA (5′-CAATCCCGAACGATTCTTGAAGG-3′) and 175 ng/μL ssODN *chc-1*[P892L] (5′-ACAATGCGATGGCCAAGATTTACATAGATTCCAATAATAACCTCGAGCGTTTTTTAAAAGAGAAGCCCTACTACGATAGCAAAGTTGTTGGAAAG-3′), or 350 ng/μL *chc-1*[L1049P] crRNA (5′-GGCCGATAGAACTCGTGTGA-3′) and 175 ng/μL ssODN *chc-1*[L1049P] (5′-ATTATTTTAGAAAACATATTCGATCAATCCACTTTCAGGCAGACAGAACACGAGTCATGGAATATATCCAGAAACCTGATAATTATGATGCTCCAGATATTGCTAACATTGCTATAAC-3′). To isolate worms harboring the *chc-1* c.2675C > T nucleotide change, PCR amplification was performed using a single forward primer (5′-CTGCGATCGTCATAACATGG-3′) and two reverse primers annealing with the WT (5′-CTACGATGAACATTCTCTTCG-3′) or the modified (5′-GTTCTCTTTTAAAAAACGCTCG-3′) sequence. To isolate worms harboring the *chc-1* c.3146 T > C nucleotide change, PCR amplification was performed using a single forward primer (5′-CATCTCTGTGACTGTTAAGGC-3′) and two reverse primers annealing with the WT (5′-CCAGACATCTGACTGGTTGCAC-3′) or the modified (5′-GGTTTCTGGATATATTCCATG-3′) sequence. Genotyping was confirmed by Sanger sequencing. Two independent lines carrying the p.P892L mutation were generated; both were out-crossed three times to the N2 strain in order to remove potential off-targets. These lines showed an equivalent phenotype and were designated as *chc-1*(*pan1*[P892L]).

The WT and mutant *chc-1* cDNAs were subcloned into the pBG-GY152 vector to be under the control of the pan-neuronal *rgef-1* promoter, and were injected at 2 ng/μL to generate multi-copy extrachromosomal arrays. Higher concentrations of the transgene (5 and 10 ng/μL) were toxic causing embryonic lethality and larval arrest. The pJM371 plasmid (p*elt-2*::*NLS*::*GFP*) (a gift from J.D. McGhee, University of Calgary), driving GFP expression in intestinal cell nuclei, was used as co-injection marker (30 ng/μL). Transgenic lines overexpressing the WT or mutant *chc-1* alleles were used in aldicarb assay to test the DN effect of the p.P892L substitution. Transgenic lines overexpressing WT *chc-1* were crossed with *chc-1*[P892L] animals for rescue experiments. For each genotype, three independent lines/clones were isolated and tested in drug-sensitivity and locomotor assays.

Sensitivity to aldicarb, levamisole and PTZ (Sigma-Aldrich) was determined as described (Mahoney et al., 2006; Thapliyal and Babu, 2018; Wong et al., 2018; Di Rocco et al., 2022). BSR assay was performed in young adults as detailed by Chase et al. (2004). Slowing on the bacterial lawn was calculated by dividing the difference between the locomotion rate on and off food by the locomotion rate off food (Lanzo et al., 2018). SWIP assay was carried out by measuring the thrashing rate of L4 larvae (McDonald et al., 2007). Phenotypic analyses were conducted at a Leica MZ10F (Leica Microsystems) and a Nikon SMZ18 (Nikon Europe) stereomicroscope.

For UHPLC–MS/MS analysis, ~5,000 L3 larvae were collected. Larvae were washed three times with M9 buffer, pelleted, and solubilized with 0.2 mL 0.5% formic acid in acetonitrile added with 10 ng of isoproterenol (internal standard), and then sonicated three times for 15 s. To prevent acetylcholine breakdown, a second sample of worms was exposed to 1 mM aldicarb. Samples were kept on ice and 5 and 10 μL of lysate were used to measure protein content by BCA assay (Thermo Scientific). The lysate were dried, solubilized with 100 μL of 0.1% formic acid and methanol (90:10, v/v), and 10 μL was injected for quantification of gamma-amino butyric acid (GABA), glutamic acid (Glu), acetylcholine (ACh), dopamine (DA), and cAMP. UHPLC–MS/MS was performed following the protocol by Bergh et al. (2016) with minor changes. Chromatography was carried out using the Acquity HSS T3 column (Waters) and a linear gradient elution with 0.1% formic acid (mobile phase A) and methanol (mobile phase B). The gradient profile is reported in Supplementary Table S2. The flow rate was kept constant at 0.4 mL/min. MS was performed with Electrospray ionization (ESI) in a positive mode. ESI conditions follow: voltage = 0.6 kV, desolvation T = 450°C, source T = 150°C, cone gas flow rate = 60 L/h, desolvation gas flow rate = 1,000 L/h, collision gas flow rate = 0.11 mL/min. Parameters are given in Supplementary Table S3. UHPLC–MS/MS data have been deposited in the Figshare Dryad Digital Repository.^3^

Locomotion parameters were analyzed quantitatively using an automated tracking system, as previously detailed (Di Rocco et al., 2022). As defined in Hart (2006), body bends are counted as events in which the region behind the pharynx returns to its initial position after reaching maximum curvature in the opposite direction from the starting position; reversals are defined as backward movements equal to at least one-fifth of the animal’s length, corresponding to the length of the pharynx.

To monitor yolk uptake, we crossed *chc-1*[P892L] animals with the transgenic strain *pwIs23 (vit-2::GFP)* expressing GFP-fused YP170, a yolk protein. To visualize SVs, we used the *jsIs219* ((p*SY3*) *sng-1*::*GFP* + *rol-6*(*su1006*)) strain crossed with engineered worms. These animals express GFP-tagged synaptogyrin, an integral membrane protein associated with presynaptic vesicles (Mullen et al., 2012). Microscopy observations were conducted at the Eclipse Ti2-E microscope (Nikon Europe) equipped with DIC optics on live animals mounted on 2% agarose pads containing 10 mM sodium azide as anesthetic.

Learning assay was performed following conditioning with high doses of benzaldehyde, as previously described (Nuttley et al., 2001), with some modifications. Ethanol-diluted benzaldehyde solutions (1 and 15%) were freshly prepared. Synchronized animals were collected and centrifuged at 1,700 rpm for 3 min. Three washes with M9 buffer were performed to remove bacteria before placing animals in the conditioning plates (3.5 cm dishes containing 2 mL of the assay medium: 2% agar, 5 mM KPO_4_ buffer pH 6.0, 1 mM CaCl_2_, 1 mM MgSO_4_). Worms were placed in conditioning or mock plates without food. Before conditioning, two agar plugs were placed onto the lid of the plates. Then, 1 μL of 100% ethanol or 15% benzaldehyde was added to the corresponding plug. Worms were conditioned for 3 h. Single-worm chemotaxis assays were performed using 1% benzaldehyde, as previously described (Lanza et al., 2021). The chemotaxis score was calculated as the sum of scores of the sectors through which the animal had traveled. The score ranges between +6 and −6. A positive value indicates attraction; a negative value indicates repulsion.

Genotype blinding was used for all experiments except for UHPLC–MS/MS analysis. Statistical differences were calculated using the GraphPad Prism 8.4.2 software.

## Data availability statement

The original contributions presented in this study are included in the article/Supplementary material; UHPLC–MS/MS data have been deposited in the Figshare Dryad Digital Repository (https://figshare.com/s/84aeb8323ad517a5f07a). Further inquiries can be directed to the corresponding authors.

## Ethics statement

The studies involving human participants were reviewed and approved by the Sapienza University of Rome. The patients/participants provided their written informed consent to participate in this study.

## Author contributions

SM, MT, and VL contributed to conception and design of the study. LP, MR, EM, FT, GO, EL, and LB performed *C. elegans* studies and the data analysis. VM and SP performed *in vitro* studies and the data analysis. SM, SG, and ES wrote the first draft of the manuscript. FN, FM, and VF wrote sections of the manuscript. All authors contributed to manuscript revision, read, and approved the submitted version.

## Funding

This work was supported by the Istituto Superiore di Sanità (Ricerca Indipendente 2020-2022_ISS20-0ab01a06bd2a to SM) and Italian Ministry of Health (CCR-2017-23669081, RCR-2022-23682289, and 5x1000_2019 to MT). MR was recipient of a research fellowship from the Associazione Famiglie GNAO1 APS.

## Conflict of interest

EL and VF were employed by D-Tails s.r.l.

The remaining authors declare that the research was conducted in the absence of any commercial or financial relationships that could be construed as a potential conflict of interest.

## Publisher’s note

All claims expressed in this article are solely those of the authors and do not necessarily represent those of their affiliated organizations, or those of the publisher, the editors and the reviewers. Any product that may be evaluated in this article, or claim that may be made by its manufacturer, is not guaranteed or endorsed by the publisher.

## Footnotes

^1^ https://www.ncbi.nlm.nih.gov/homologene/

^2^ https://gnomad.broadinstitute.org/

^3^ https://figshare.com/s/84aeb8323ad517a5f07a
