# Supplementary material for: The recurrent pathogenic Pro890Leu substitution in CLTC causes a generalized defect in synaptic transmission in Caenorhabditis elegans
Source: Front Mol Neurosci. 2023 May 31;16:1170061. doi: 10.3389/fnmol.2023.1170061 (PMC10264582; doi:10.3389/fnmol.2023.1170061)
Supplement: Supplementary file 2 [file Data_Sheet_1.pdf]

Supplementary Material

The recurrent pathogenic Pro890Leu substitution in *CLTC* causes a generalized defect in synaptic transmission in *Caenorhabditis elegans*

Luca Pannone, Valentina Muto, Francesca Nardecchia, Martina Di Rocco, Emilia Marchei, Federica Tosato, Stefania Petrini, Giada Onorato, Enrico Lanza, Lucia Bertuccini, Filippo Manti, Viola Folli, Serena Galosi, Elia Di Schiavi, Vincenzo Leuzzi, Marco Tartaglia*, Simone Martinelli*

***Correspondence:** Simone Martinelli, simone.martinelli@iss.it or Marco Tartaglia, marco.tartaglia@opbg.net

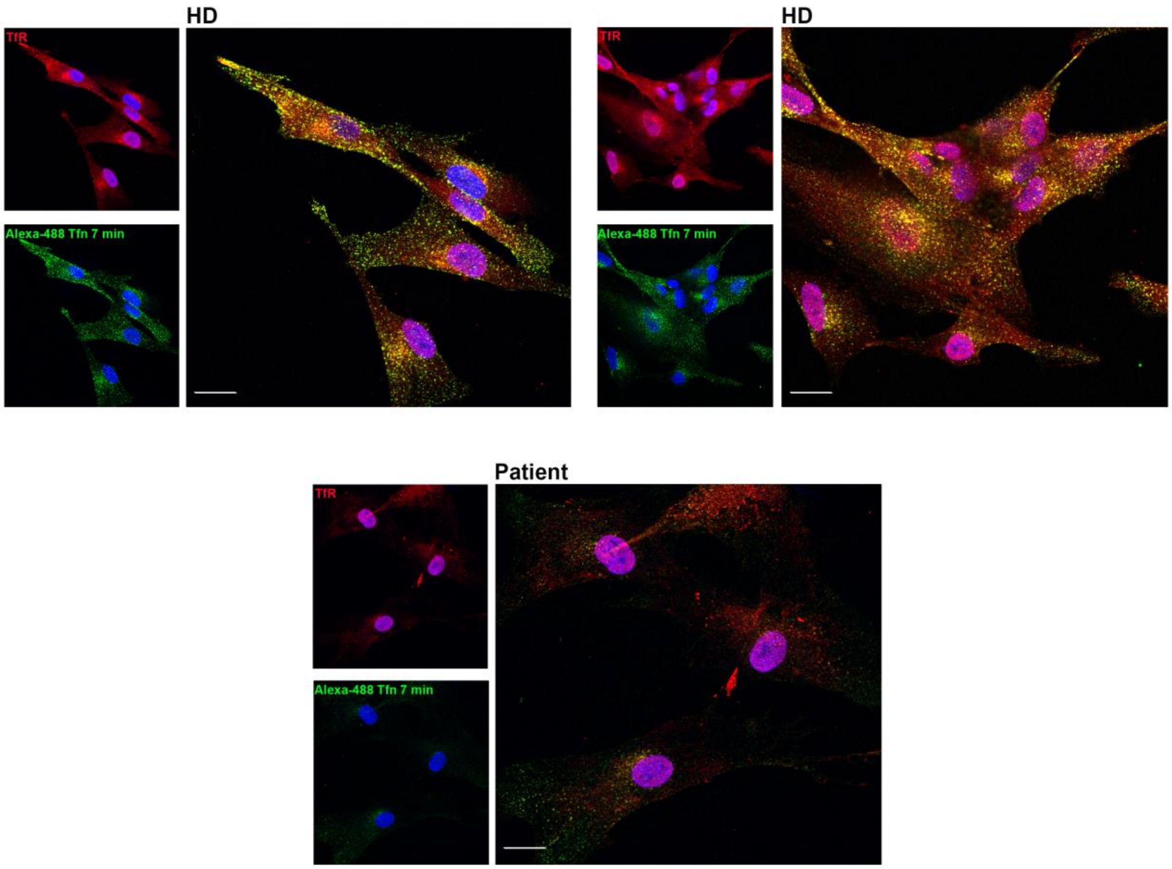

Supplementary Figure S1. Detrimental effect of the p.P890L amino acid substitution on intracellular trafficking. Confocal microscopy analysis shows aberrant uptake of transferrin (Tf) in patient's cells. Fibroblasts obtained from the patient and two different healthy donors were serum starved, incubated with Alexa Fluor 488-conjugated Tf, and stained with anti-Tf receptor (TfR) antibody and Alexa Fluor 594 goat anti-mouse secondary antibody (red). Scale bar, 20 μm.

```
H.sapiens   777   VCDRFDFVHDLVLYLYRNNLQKYIEIYVQKVNPSRLPVVIGGLLDVDCSEDVIKNLILVV  836
                  VCDR + VHDLVLYLYRN LQKYIE++VQKVN +RLP+V+G LLDVDCSED IK LI+
C. elegans 779    VCDRHNMVHDLVLYLYRNQLQKYIEVFVQKVNAARLPIVVGALLDVDCSEDAIKQLIINT  838

H.sapiens   837   RGQFSTDELVAEVEKRNRLKLLLPWLEARIHEGCEEPATHNALAKIYIDSNNNPERFLRE  896
                  RG+F  DELV EVEKRNRLKLL  WLE++I EG   + ATHNA+AKIYIDSNNNPERFL+E
C. elegans 839    RGKFDIDELVEEVEKRNRLKLLNHWLESKIQEGATDAATHNAMAKIYIDSNNNPERFLKE  898

H.sapiens   897   NPYYDSRVVGKYCEKRDPHLACVAYERGQCDLELINVCNENSLFKSLSRYLVRRKDPELW  956
                  NPYYDS+VVGKYCEKRDPH A ++YERGQCD ELINVCNENSLFK+L+RYLV+R+D  LW
C. elegans 899    NPYYDSKVVGKYCEKRDPHYAFLSYERGQCDAELINVCNENSLFKNLARYLVKRRDFTLW  958

H.sapiens   957   GSVLLESNPYRRPLIDQVVQTALSETQDPEEVSVTVKAFMTADLPNELIELLEKIVLDNS  1016
                    VL E N +RR LIDQVVQTALSETQDPE++SVTVKAFM ADLPNELIELLEKIVLDNS
C. elegans 959    EQVLNEENVHRRQLIDQVVQTALSETQDPEDISVTVKAFMAADLPNELIELLEKIVLDNS  1018

H.sapiens   1017  VFSEHRNLQNLLILTAIKADRTRVMEYINRLDNYDAPDIANIAISNELFEEAFAIFRKFD  1076
                   FSEHRNLQNLLILTA++ADRTRVMEYI +LDNYDAPDIANIAI++EL+EEAFAIF+KFD
C. elegans 1019   AFSEHRNLQNLLILTAMRADRTRVMEYIQKLDNYDAPDIANIAITSELYEEAFAIFKKFD  1078

H.sapiens   1077  VNTSAVQVLIEHIGNLDRAYEFAERCNEPAVWSQLAKAQLQKGMVKEAIDSYIKADDPSS  1136
                  VN+SA+ VLIE++ NLDRAYEFAE+CN+  VW+ LAKAQLQ+ +VKEA+DS+IKADDP +
C. elegans 1079   VNSSAINVLIENVNNLDRAYEFAEKCNQSDVWASLAKAQLQQNLVKEAVDSFIKADDPGA  1138
```

Supplementary Figure S2. Amino acid sequence alignment of human CLTC and the *C. elegans* CHC-1 orthologue protein (https://www.ncbi.nlm.nih.gov/homologene). Alignment encompasses the two affected residues characterized in the present study (*h*Pro^{890}/*Ce*Pro^{892} and *h*Leu^{1047}/*Ce*Leu^{1049}), which are highlighted in yellow/bold and included in a black box. Identity and conservation (+) of individual residues is reported in the middle row.

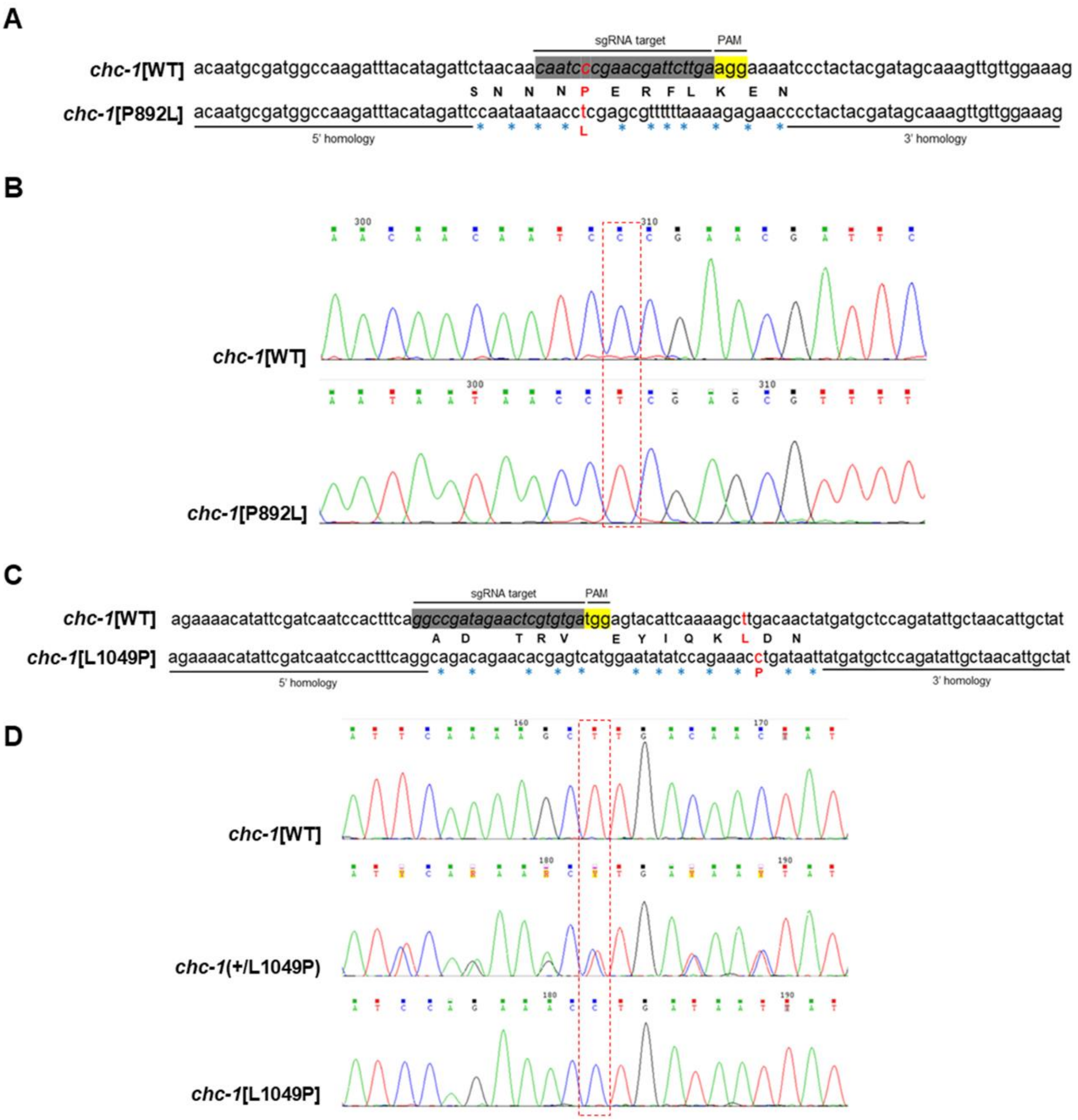

Supplementary Figure S3. Gene editing approach and validation through Sanger sequencing. (**A, C**) Gene editing design for the c.2669C>T (p.Pro892Leu) and c.3146T>C (p.Leu1049Pro) substitutions. The modified bases and the resulting amino acid changes are highlighted in red. Silent substitutions (cyan asterisks) have been introduced to prevent Cas9 re-cutting and to facilitate PCR genotyping. (**B, D**) Chromatograms showing the presence of the index mutations.

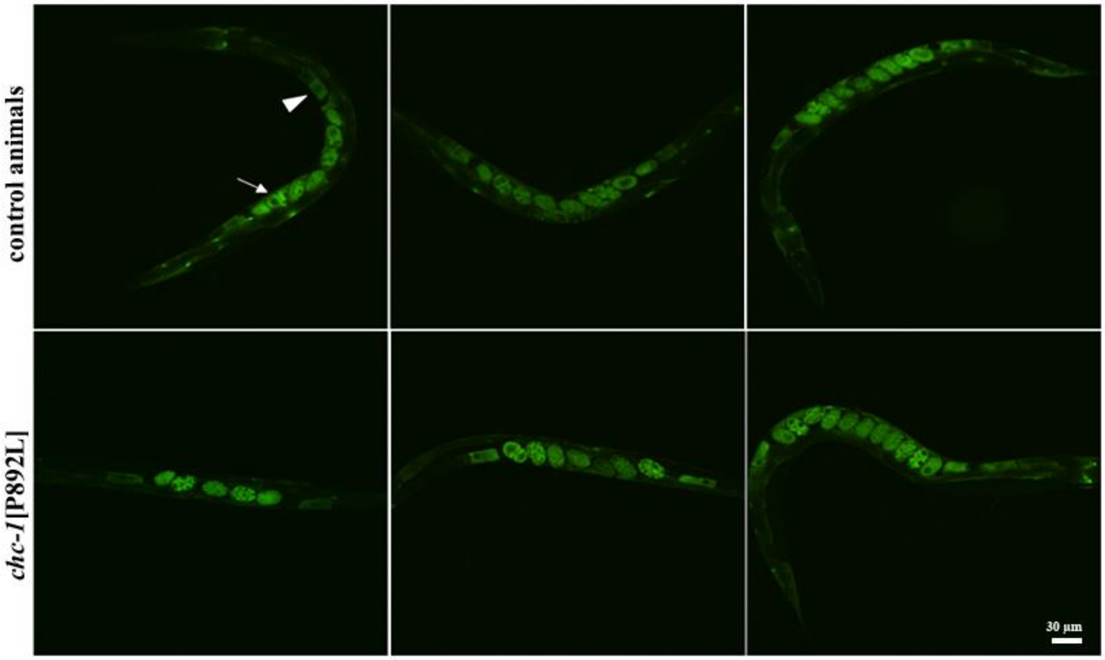

Supplementary Figure S4. Yolk uptake by oocytes of adult hermaphrodites is not affected in *chc-1*[P892L] animals. YP170-GFP is synthesized in the intestine and secreted into the body cavity, from which it is taken up by oocytes. In both wild-type and mutant animals, the yolk is efficiently endocytosed by the oocytes (arrowhead) and, after fertilization, is found within the embryos (arrow). Scale bar, 30 µm.

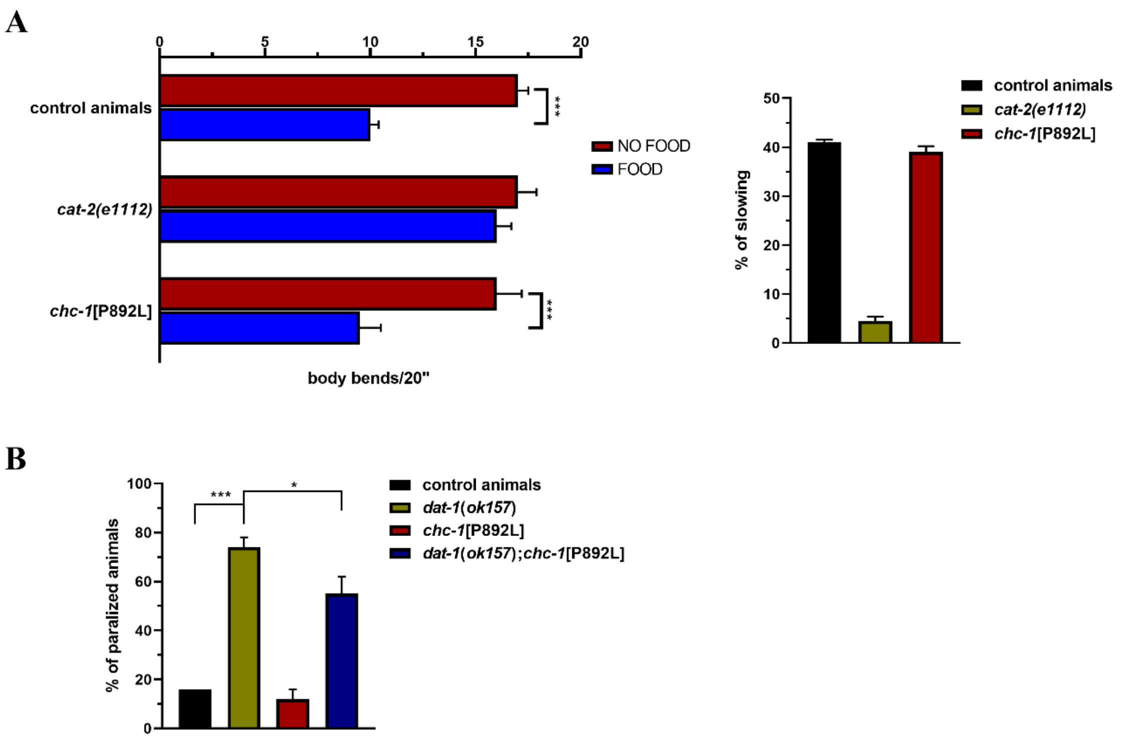

Supplementary Figure S5. Dopamine signaling is slightly affected in *chc-1*[P892L] animals. (**A**) BSR assay. The number of body bends counted for five consecutive 20 sec-intervals in the presence (red) or absence (blue) of food are shown (left panel). No difference between *chc-1*[P892L] and control animals was observed, whereas *cat-2(e1112)* worms did not show any slowdown in the presence of food (***p < 0.0001; unpaired t-test with Welch's correction). The percentage of slowing calculated by dividing the difference between the locomotion rates on and off food by the locomotion rate off food is also reported (right panel). Control animals, n=20; *cat-2(e1112)*, n=20; *chc-1*[P892L], n=30. (**B**) SWIP assay. Bars indicate the percentage of paralyzed animals after 10 minutes swimming. *chc-1*[P892L] worms rescued, in part, the phenotype observed in *dat-1* knock-out mutants, indicating slightly reduced dopamine release (*p < 0.01, ***p < 0.0001; one-way Anova with Bonferroni correction). One hundred animals for each genotype were tested. Data represent means ± SD of at least three independent experiments.

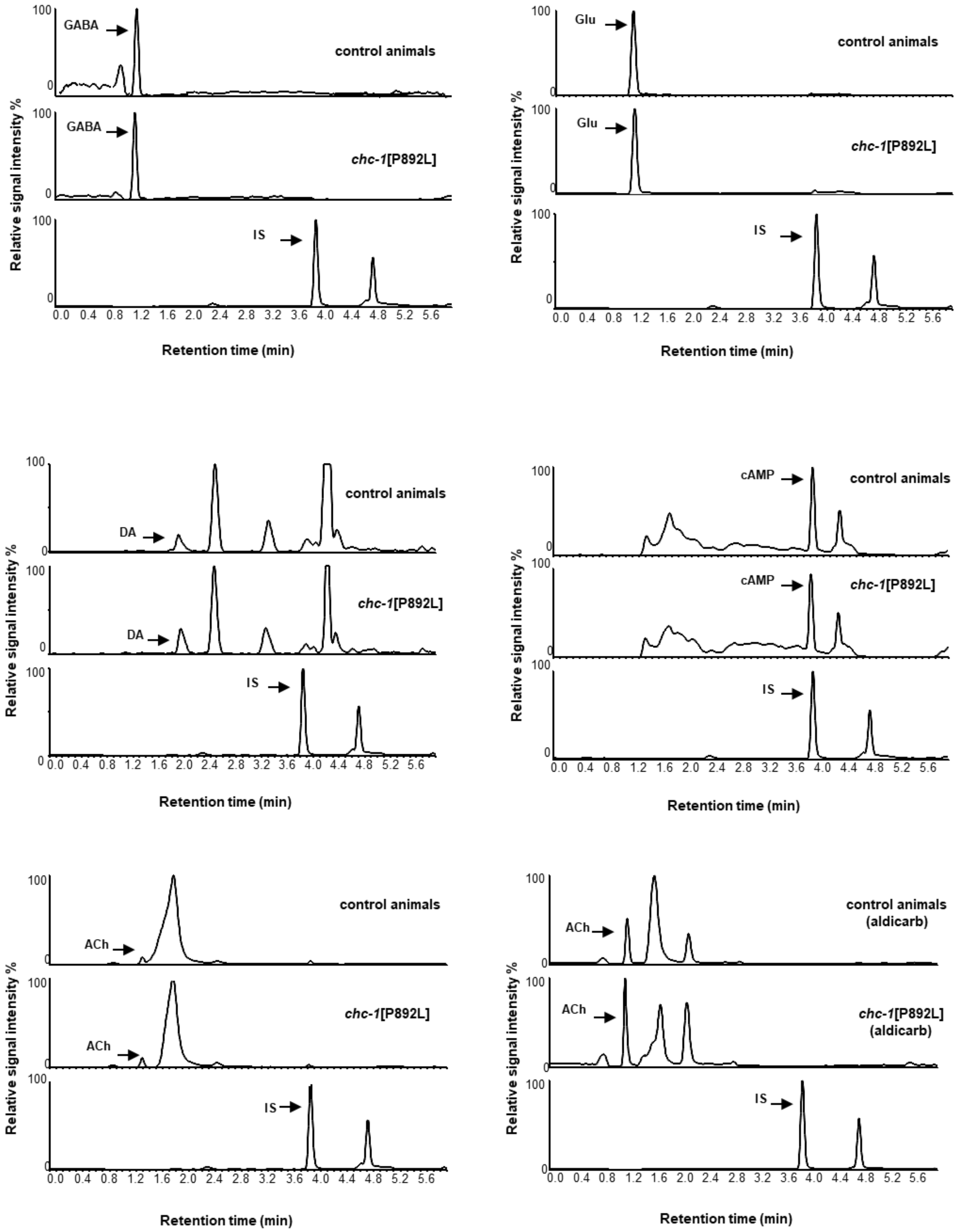

Supplementary Figure S6. UHPLC–MS/MS chromatograms. MRM traces of gamma-amino butyric acid (GABA), glutamic acid (Glu), acetylcholine (ACh), dopamine (DA), cyclic adenosine monophosphate (cAMP), and isoproterenol (internal standard, IS) in control and *chc-1*[P892L] animals. For ACh quantification, nematodes were treated with aldicarb, an acetylcholinesterase inhibitor, in order to avoid ACh degradation. The quantifier mass transition (see Table 2) of neurotransmitters and the internal standard are shown.

Supplementary Table S1. WES statistics and data output.

WES enrichment kit	Illumina Nextera
Sequencing platform	NextSeq500
Target regions coverage >10x	93%
Target regions coverage >20x	88%
Average depth on target	86x
Total number of high-quality variants	107,556
Low frequency variants affecting CDS or splice site/regions[1]	248
Putative disease associated genes[2]:	
- candidate genes (autosomal dominant)	1[3]
- candidate genes (autosomal recessive)	0

[1]High-quality, rare/private (gnomAD MAF < 0.1%; *in house* database MAF < 1%), functionally relevant variants within coding exons and splice regions (-3/+8).

[2]High-quality, rare/private, functionally relevant variants with both CADD phred > 20.0 and M-CAP > 0.025 affecting OMIM genes.

[3]*CLTC* (c.2669C>T, p.Pro890Leu, CADD = 29.6, M-CAP = 0.126).

Supplementary Table S2. Mobile-phase gradient in UHPLC-MS/MS separation.

Time (min)	% mobile phase A	% mobile phase B
0	99.0	1.0
0.50	99.0	1.0
1.20	90.0	10.0
1.50	80.0	20.0
1.90	75.0	25.0
2.80	40.0	60.0
3.20	20.0	80.0
3.25	0.0	100.0
4.25	0.0	100.0
5.00	99.0	1.0
10.00	99.0	1.0

Supplementary Table S3. UHPLC–MS/MS parameters for the MRM acquisition mode (quantification and confirmation).

Analytes	Relative Retention time	MRM transitions					
		Quantification			Confirmation		
		m/z	CV[a]	CE[b]	m/z	CV[a]	CE[b]
GABA	0.30	103.90 > 86.80	16	8	103.90 > 68.90	16	14
Glu	0.30	148.00 > 84.00	16	14	103.90 > 101.90	16	12
ACh	0.35	146.20 > 87.00	20	14	146.20 > 60.00	20	14
DA	0.63	153.90 > 136.80	20	10	153.90 > 91.00	20	28
cAMP	1.01	330.27 > 136.05	30	22	330.27 > 119.05	30	46
Isoproterenol (IS)[c]	1.00	212.20 > 194.21	20	10			

[a]CV, cone voltage; [b]CE, collision energy; [c]IS, internal standard.

Legend of Supplementary movie S1. Time-lapse confocal imaging by XYZ stack acquisition in patient's-derived fibroblasts and control cells. XYZ stacks were acquired every 8 seconds for a total time of 10 minutes. Maximal projection of the XYZ stack is shown.
